# Disulfide cross-linked multimers of TDP-43 and spinal motoneuron loss in a TDP-43^A315T^ ALS/FTD mouse model

**DOI:** 10.1038/s41598-017-14399-5

**Published:** 2017-10-27

**Authors:** Leslie Bargsted, Danilo B. Medinas, Francisca Martínez Traub, Pablo Rozas, Natalia Muñoz, Melissa Nassif, Carolina Jerez, Alejandra Catenaccio, Felipe A. Court, Claudio Hetz, Soledad Matus

**Affiliations:** 1 Fundacion Ciencia & Vida, Santiago, 7780272 Chile; 2 Biomedical Neuroscience Institute, Faculty of Medicine, University of Chile, Santiago, Chile; 3 Geroscience Center for Brain Health and Metabolism (GERO), Santiago, Chile; 4 Program of Cellular and Molecular Biology, Institute of Biomedical Sciences, University of Chile, Santiago, Chile; 5 Center for Integrative Biology, Faculty of Sciences, Universidad Mayor, Santiago, Chile; 6 Buck Institute for Research on Aging, Novato, CA 94945 USA; 7 Department of Immunology and Infectious diseases, Harvard School of Public Health, Boston, MA USA; 8 Neurounion Biomedical Foundation, Santiago, Chile

## Abstract

Tar DNA binding protein 43 (TDP-43) is the principal component of ubiquitinated protein inclusions present in nervous tissue of most cases of both amyotrophic lateral sclerosis (ALS) and frontotemporal dementia (FTD). Previous studies described a TDP-43^A315T^ transgenic mouse model that develops progressive motor dysfunction in the absence of protein aggregation or significant motoneuron loss, questioning its validity to study ALS. Here we have further characterized the course of the disease in TDP-43^A315T^ mice using a battery of tests and biochemical approaches. We confirmed that TDP-43 mutant mice develop impaired motor performance, accompanied by progressive body weight loss. Significant differences were observed in life span between genders, where females survived longer than males. Histopathological analysis of the spinal cord demonstrated a significant motoneurons loss, accompanied by axonal degeneration, astrogliosis and microglial activation. Importantly, histopathological alterations observed in TDP-43 mutant mice were similar to some characteristic changes observed in mutant SOD1 mice. Unexpectedly, we identified the presence of different species of disulfide-dependent TDP-43 aggregates in cortex and spinal cord tissue. Overall, this study indicates that TDP-43^A315T^ transgenic mice develop key features resembling key aspects of ALS, highlighting its relevance to study disease pathogenesis.

## Introduction

Amyotrophic lateral sclerosis (ALS) is a progressive and lethal degenerative disorder that affects motoneurons in the brain and spinal cord. ALS patients develop paralysis of voluntary muscles, accompanied by an exaltation of tendon reflexes, muscle weakness, spasticity and atrophy^1,2^. Frontotemporal dementia (FTD) is the second most common neurodegenerative disease in patients over 65 years old after Alzheimer’s disease, and it is characterized by the progressive failure of frontal and temporal lobes of the brain^3,4^. Clinical manifestations of FTD include changes in behavior and personality, accompanied by language alterations. Importantly, a fraction of FTD patients also develop motoneuron diseases, including ALS^5^, suggesting that they are part of a unique pathological spectrum^6^.

Tar DNA binding protein 43 (TDP-43) was identified as the major constituent of the ubiquitinated protein inclusions present in brain and spinal cord of most cases of ALS and frontotemporal lobar degeneration with ubiquitinated inclusions (FTLD-U), a type of FTD^7,8^. Under normal conditions, TDP-43 locates in the nucleus with functions related to transcriptional regulation, mRNA stability, mRNA traffic, pre-mRNA splicing and miRNA biogenesis^9–13^. Interestingly, the subcellular distribution of TDP-43 is altered in ALS/FTD, with the presence of cytoplasmic inclusions containing ubiquitinated and phosphorylated forms of the protein, in addition to proteolytic fragments^8,14^. Mutations in the TDP-43 gene are also described in familial and sporadic cases of ALS (revised in ref.^15^). TDP-43 pathogenesis is proposed to emerge due to a loss-of-function in the nucleus combined with a “gain of toxic” activity in the cytoplasm^15^. The histopathological features related to TDP-43 have also been found in sporadic and familial cases of ALS and FTD^8^. Due to the failure of most translational efforts derived from studies using mutant SOD1 mice (present in less than 2% of ALS cases), it is essential to develop and validate new complementary models to study ALS.

Several animal models to study TDP-43 pathogenesis have been developed in mouse and rats with a variety of phenotypes and histopathological features (reviewed in ref.^16^). For example, the overexpression of human wild-type TDP-43 (TDP-43^WT^) in neurons causes motor impairment^17–19^. Transgenic mice expressing TDP-43^WT^ in the hippocampus, cortex and striatum develop learning and memory deficits, as well as altered motor control ^20^. Gliosis is also detected in most mouse models overexpressing TDP-43^WT^ ^17,19–21^. In these models, ubiquitin positive cytoplasmic inclusions can be found in spinal cord motoneurons and brainstem neurons. Other groups have developed inducible transgenic mice to study TDP-43 pathogenesis. The expression of a truncated TDP-43 form, lacking the nuclear localization, leads to neuronal loss in certain forebrain regions, corticospinal tract degeneration and also motor spasticity^22^. Motor and cognitive alterations have been also found in mice expressing TDP-43^A315T^ mutant at 3 months of age^23^. All these studies suggest that different patterns of TDP-43 expression result in either motor dysfunctions or behavioral alterations depending on the tissue and levels of transgene expression, generating a phenotype resembling FTD-ALS. However, none of the models described so far are optimal to study ALS because of the apparent absence of motoneurons degeneration.

One of the first mouse models described to study TDP-43 biology expresses the ALS-linked mutation A315T controlled by mouse prion promoter^24^. The original report indicated that this model undergoes a selective loss of upper motoneurons, correlating with cortical gliosis^24,25^. This transgenic line also showed gait alterations^24^ and loss of muscle strength^17^. Although TDP-43^A315T^ transgenic mice developed motor problems, the authors failed to identify lower motoneuron loss or signs of TDP-43 aggregation^24^, questioning its possible use to study ALS pathophysiology.

Here we have further characterized the TDP-43^A315T^ transgenic mice using a battery of motor and biochemical assays. As reported before, we confirmed the gender differences in the progression of motor problems, observing that females TDP-43^A315T^ mice live longer than males, despite having similar duration of the symptomatic stage. Remarkably, we observed the progressive appearance of coordination and motor problems. Importantly, histopathological analysis using serial sections revealed the occurrence of extensive neuronal loss in the ventral horn of the spinal cord, in the same population affected in a well described mutant SOD1 mice. Accordingly, total axons at the ventral root of mutant TDP-43 mice were decreased, accompanied by an augmented number of degenerating axons. Moreover, we observed clear signs of microglial and astrocyte activation, to a similar extent as mutant SOD1 transgenic mice. Finally, using different biochemical approaches, we demonstrate that spinal cord and cortical brain tissue contain distinct species of disulfide-crosslinked aggregates of TDP-43 that were sensitive to reducing agents. Taken together, our study suggests that TDP-43^A315T^ transgenic mice develop important features linked to ALS and FTD that were previously unrecognized, highlighting its possible use to study disease pathogenesis and the development of future therapeutic interventions.

## Results

### Differential survival of male and female TDP-43^A315T^ transgenic mice

**Figure 1:**
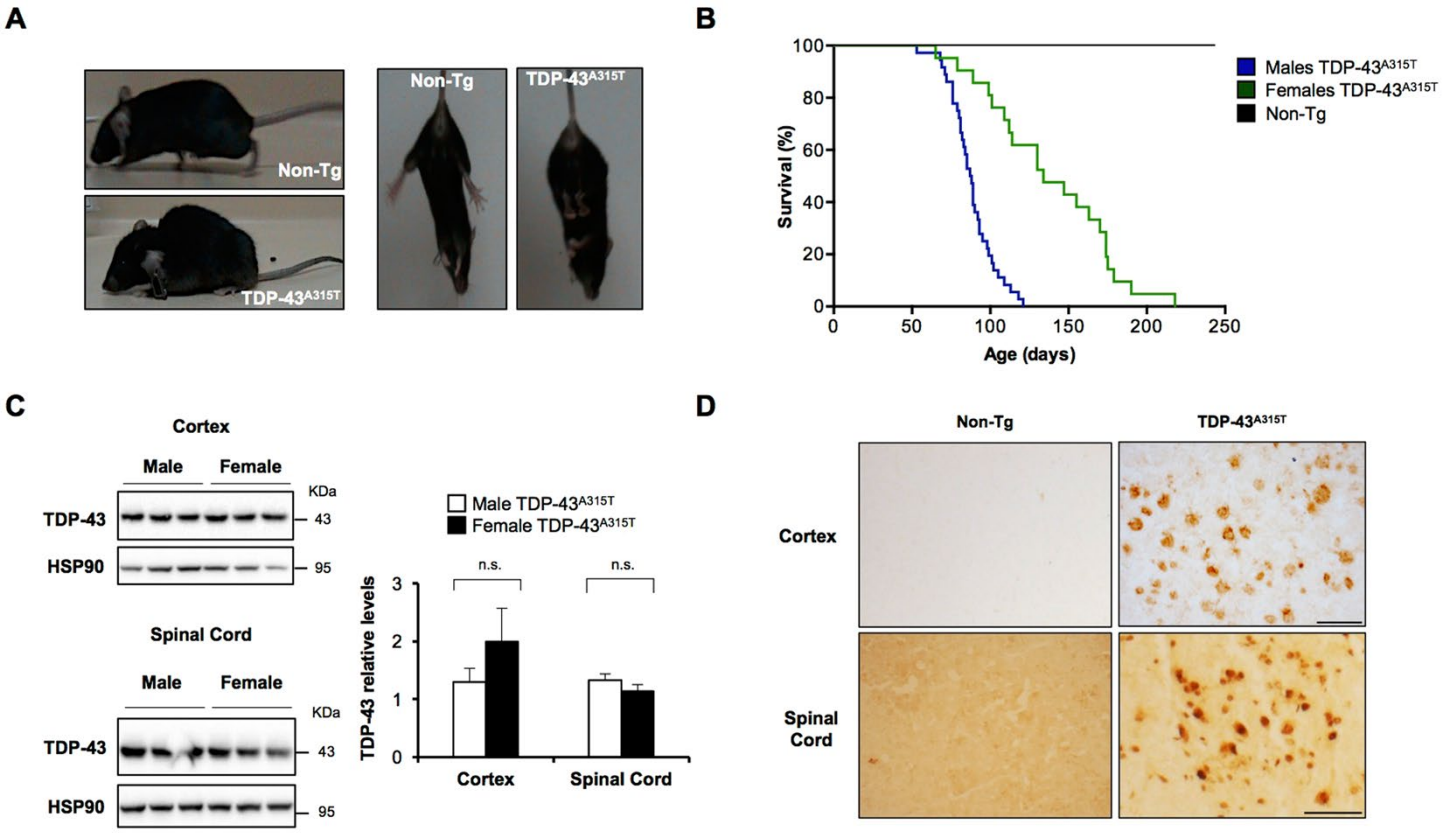
Survival of transgenic TDP-43^A315T^ male and female mice. (**A**) Images showing the disease phenotype (spinal cord curvature and hind limbs fall) of a TDP-43^A315T^ mouse in symptomatic stage. As control, a Non-Tg littermate of the same age (179 days, female) is shown. (**B**). Survival of male (blue line, N = 36) and female (green line, N = 21) TDP-43^A315T^ transgenic mice was monitored over time. All Non-Tg animals survived beyond 200 days (black line, N = 83). (**C**) TDP-43 protein levels were analyzed in brain cortex (upper panel) and spinal cord (lower panel) extracts of male (N = 3) and female (N = 3) TDP-43^A315T^ mice using Western blot. Quantification (right panel) was performed after normalization with HSP90 as a loading control. Statistical analysis was performed using non-parametric Mann-Whitney test. n.s., *p* > 0.05. (**D**) FLAG detection by immunohistochemistry approach in brain cortex and spinal cord tissue of TDP-43^A315T^ mice and Non-Tg littermates in adult stage. Length bar upper panel: 50 μm, length bar lower panel: 100 μm.

In order to investigate the consequences of expressing mutant TDP-43 in mice, we further characterized a transgenic line which overexpresses TDP-43^A315T^ under the control of the murine prion protein promoter^24^. Visual observation of disease progression indicated the occurrence of kyphosis, abnormal hind limb-clasping reflex, ruffling fur, and hunched posture at terminal stages (Fig. 1A). Survival analysis of a large cohort of animals showed a differential effect of mutant TDP-43 expression in life span between genders as reported before^26^ (Fig. 1B). Male mice presented a mean survival of 88 ± 2 days, whereas female animals died at 138 ± 9 days.

As control experiments, we determined the expression levels of TDP-43 in male and female mice. Analysis of tissues lysates obtained from TDP-43^A315T^ transgenic animals at end-stage indicated similar overexpression levels of TDP-43 in both frontal cortex and spinal cord of male and female animals (Fig. 1C). A near 3-fold overexpression levels were observed when compared with the endogenous protein of non-transgenic animals (not shown). Overexpression of TDP-43 was also confirmed by histology of cortex and spinal cord tissue (Fig. 1D and Figs S1 and S2).

### Motor alterations in TDP-43^A315T^ transgenic mice

**Figure 2:**
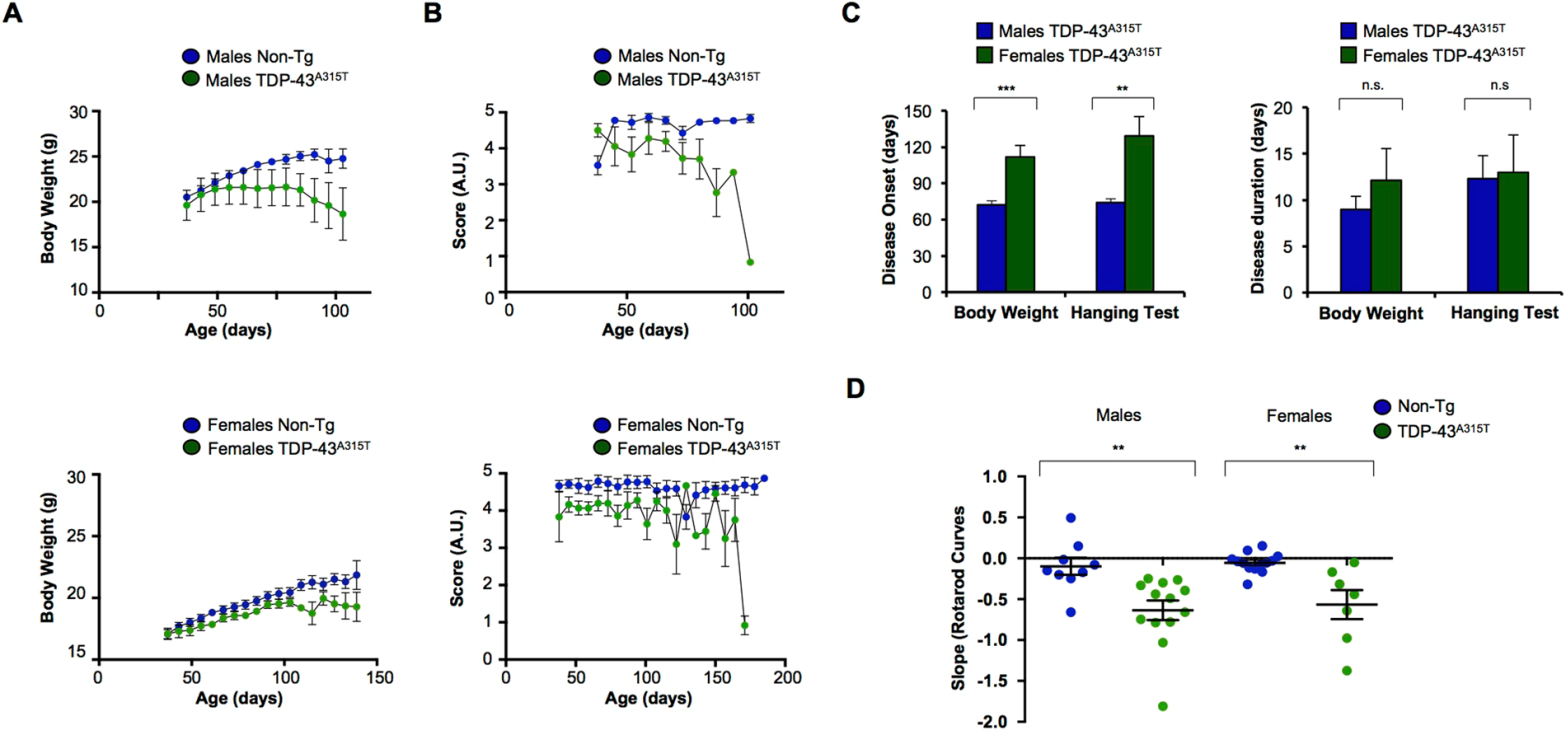
Disease progression of TDP-43^A315T^ transgenic mice. (**A**) Body weight was monitored over time in TDP-43^A315T^ transgenic mice and non-transgenic littermates, and analyzed by gender. This parameter was monitored twice a week, starting at 37 days of age. Males Non-Tg (N = 11, blue circles), males TDP-43^A315T^ (N = 16, green circles), Non-Tg females (N = 12, blue circles), TDP-43^A315T^ females (N = 9, green circles). (**B**) Analysis of motor performance over time measured by the Hanging test. As for the analysis of body weight parameter, monitoring was performed on TDP-43^A315T^ transgenic mice and non-transgenic littermates, analyzed by gender. Scores were assigned with arbitrary units (U.A.) as described in materials and methods. Males Non-Tg (N = 8, blue circles), males TDP-43^A315T^ (N = 6, green circles), females Non-Tg (N = 9, blue circles), females TDP-43^A315T^ (N = 4, green circles). (**C**) Average disease onset (left panel) and duration (right panel) was determined in TDP-43^A315T^ transgenic animals using body weight as a physiological parameter and Hanging test as a motor performance parameter. Comparison between groups with unpaired Student’s t test. ***p < 0.0001, **0.001 < p < 0.01. Average disease duration of the animal was calculated as the time between the onset of disease signs and the day of death. Comparison between groups was performed with unpaired Student’s t test. n.s. *p* > 0.05 (**D**) Slopes of individual curves of Rota-rod (for slopes determination see supplementary material, Fig. S2) in symptomatic stage. Each dot represents one animal, males Non-Tg (N = 8, blue circles), males TDP-43^A315T^ (N = 13, green circles), females Non-Tg (N = 12, blue circles), females TDP-43^A315T^ (N = 7, green circles). Comparison between groups independently with unpaired Student’s t test. **0.001 < *p* < 0.01.

To evaluate the effects of mutant TDP-43 expression on disease progression, we monitored body weight loss and motor performance over time. We observed a sustained decline in body weight in both female and male mice, where the latter showed a more drastic phenotype (Fig. 2A). Analysis of motor coordination using the Hanging test indicated reduced score in TDP-43^A315T^ mice, observing a progressive decline in male animals whereas female animals showed a clear drop of performance at later time points (Fig. 2B). Calculation of disease onset using these two parameters (see methods for criteria) indicated that female animals develop symptoms later than male mice. Using body weight measurements (criterion of 2.5% loss) an average onset of 73 ± 3 days was obtained for male mice, whereas female animals presented a phenotype at 112 ± 9 days of age (Fig. 2C). Using the Hanging wire test, disease onset was determined as the day when the performance declined, observing an average of 74 ± 3 days and 129 ± 16 days for male and female mice, respectively (Fig. 2C). Rota-rod performance of individual TDP-43 mutant mice was highly variable (not shown). However, analysis of slope of latency of individual animals, indicated that TDP-43^A315T^ mice also suffer a clear and significant reduction of performance in the Rota-rod test (Fig. 2D and Fig. S3), confirming a progressive impairment in motor capacity. Based on the determined disease onset, we calculated disease duration. Interestingly, no differences were observed between females and males using both body weight loss and Hanging test performance analysis (Fig. 2C). Overall, our results indicate that TDP-43^A315T^ transgenic animals develop motor and coordination impairment, correlating with premature death of the animals.

### Motoneuron loss and glial activation in the spinal cord of TDP-43^A315T^ mice

**Figure 3:**
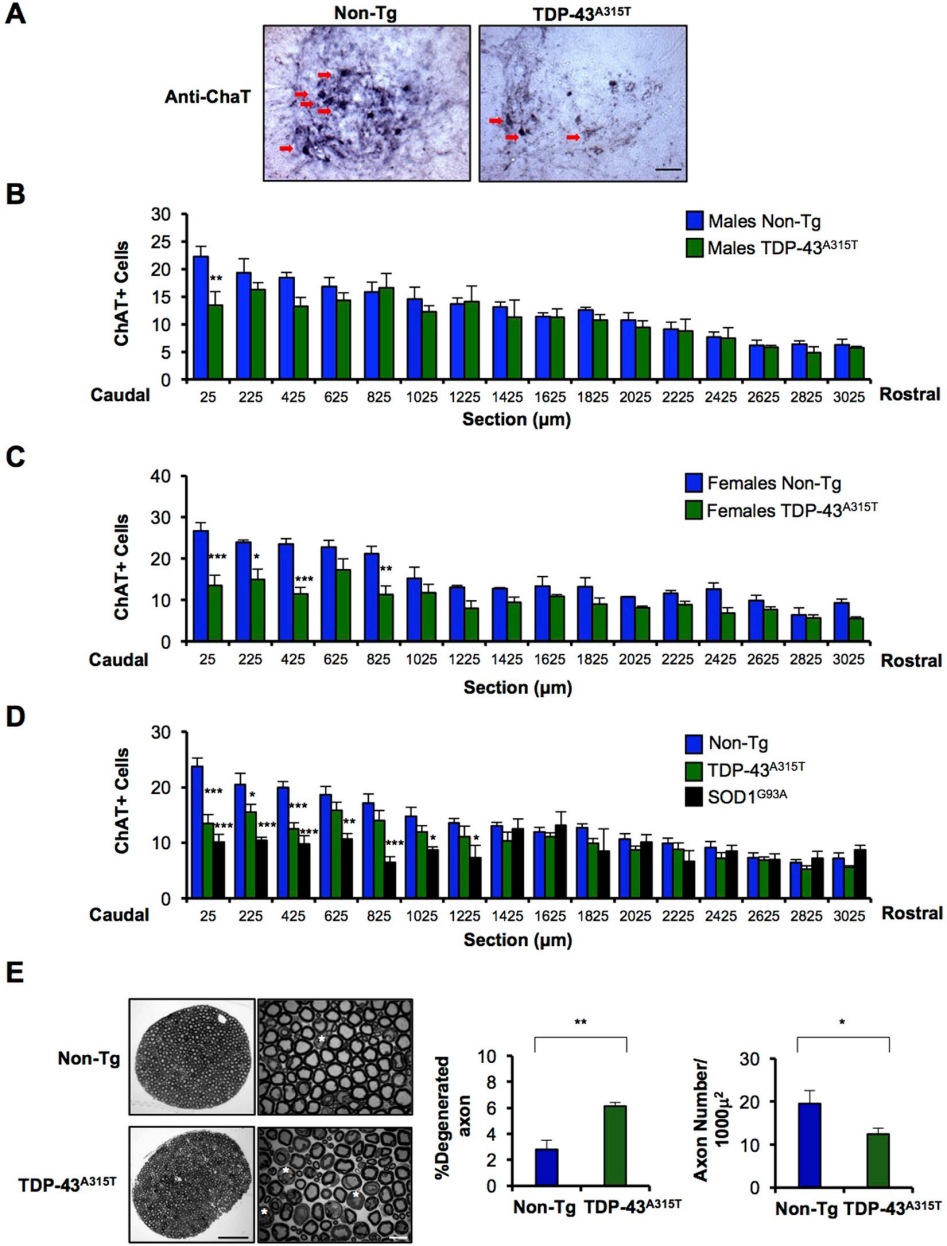
Loss of motoneurons in lumbar spinal cord of symptomatic TDP-43^A315T^ mice. (**A**) Representative images of ChAT immunohistochemistry performed in lumbar region of symptomatic TDP-43^A315T^ mice and Non-Tg littermate control for motoneuron quantification. Section 1 is shown, which correspond to the first 25 μM from L5. Bar length: 100 μm. Motoneuron detection (indicated with red arrows) was performed using anti-ChAT immunohistochemistry on symptomatic TDP-43^A315T^ transgenic mice. Quantification of motoneurons in spinal cord tissue was performed in serial sections from L5 region moving forward to the rostral side. (**B**) Comparison between male symptomatic TDP-43^A315T^ mice (N = 3) and Non-Tg littermate controls (N = 7). For statistical analysis, two-way ANOVA with Bonferroni post-tests **0.001 < *p* < 0.01. (**C**) Motoneurons count in female symptomatic TDP-43^A315T^ mice (N = 4) and respective Non-Tg controls (N = 3). For statistical analysis, two-way ANOVA with Bonferroni post-tests ***p < 0.001, *0.01 < *p* < 0.05. (**D**) Quantification of motoneurons from ChAT immunohistochemistry performed in lumbar spinal cord of symptomatic TDP-43^A315T^ mice (N = 8) and Non-Tg littermates (N = 10), adding a group of symptomatic SOD1^G93A^ mice (N = 3) as a positive control. The quantification showed here included male and female mice. For statistical analysis, two-way ANOVA with Bonferroni post-tests *0.01 < *p* < 0.05, **0.001 < *p* < 0.01, ***p < 0.0001. All the analysis described here were performed in serial sections from L5 with an interval of 200 μM between each other and plotted as average per section. (**E**) End stage TDP-43 mutant mice (N = 3) and Non-Transgenic littermate (N = 3) were sacrificed and ventral roots were obtained from L4 and L5 and stained with toluidine blue. A representative image of whole ventral root cross section from each experimental group is shown (left panel, scale bar: 100 μm). Degenerating axons are indicated with white asterisks (right panel, scale bar: 10 μm). Quantification of percentage of degenerated axons (left) and number axons per 1000 μ^2^ (right) was performed from transversal semi-thin sections of each experimental group (right panel). Comparison between groups independently with unpaired Student’s t test. **p < 0.005, *p < 0.05.

Previous characterization of TDP-43^A315T^ mice revealed a slight reduction in the number of motoneurons of the spinal cord between L3 and L5 region (near 20% of motoneuron loss)^24^. We decided to monitor the global distribution of motoneurons through the lumbar region of the spinal cord and then compare it with a classical model of ALS expressing mutant SOD1. We performed immunohistochemistry using anti-ChAT staining in serial sections of spinal cord tissue, comparing male and female mice (Fig. 3A). Serial sections covered from L5 to L2 region of the spinal cord (16 sections in total). Remarkably, we observed higher motoneuron vulnerability in females, where caudal areas presented higher percentage of neuronal loss (Fig. 3C). Surprisingly, the analysis of male TDP-43^A315T^ mice only showed significant loss of motoneurons in the first section (25 µm from L5) (Fig. 3B). Similar results were obtained when quantification of motoneurons was performed in spinal cord sections using a co-staining with anti-ChAT and anti-NeuN antibodies or after staining with Cresyl Violet. These data also suggest that the decrease in neuronal counts observed using anti-ChAT staining was not due to the regulation of ChAT expression by TDP-43 as previously suggested^27^ (Fig. S4). Importantly, comparison of histological features with SOD1^G93A^ mutant mice at the end stage indicated that the same region of the spinal cord developed motoneuron loss (Fig. 3D), suggesting that similar populations of neurons are affected in both mouse models. In agreement with these results, based on morphological criteria (See Fig. S5), we observed an increased number of degenerated axons in the ventral root (L4 and L5) of mutant TDP-43 mice (Fig. 3E). In addition, a decreased number of axons per area was observed in this model (Fig. 3E).

**Figure 4:**
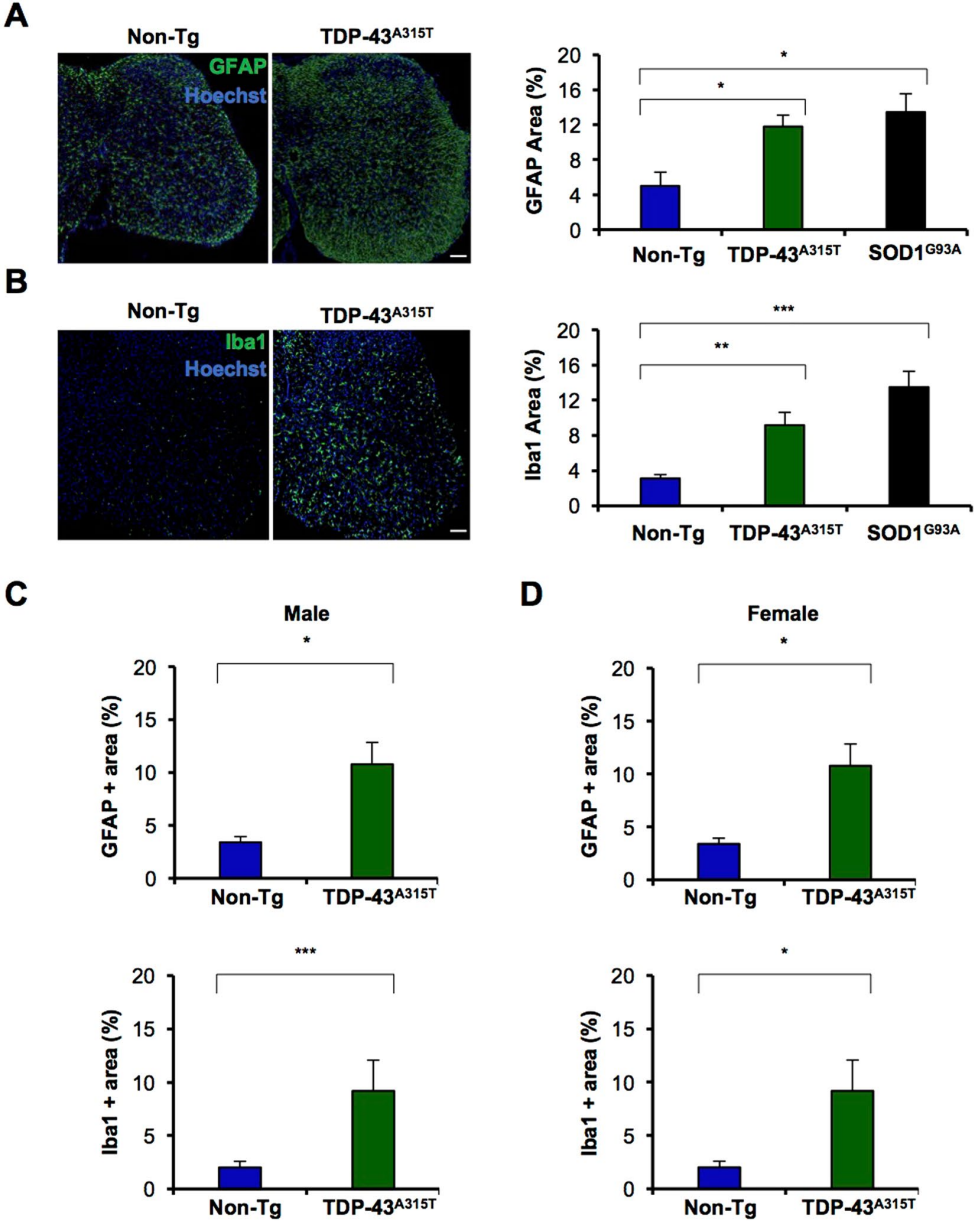
Astrogliosis and microglia activation in lumbar spinal cord of symptomatic TDP-43^A315T^ mice. (**A**) Quantification of astrogliosis was performed in serial sections using anti-GFAP immunofluorescence. Analysis of GFAP intensity on lumbar spinal cord of symptomatic TDP-43^A315T^ mice (N = 8) and Non-Tg littermates (N = 10) and representative images are shown. For statistical analysis, one-way ANOVA with Tukey post-test was performed. *0.01 < *p* < 0.05. (**B**) Quantification of microglia activation was performed as in (A), using anti-Iba1 immunofluorescence in TDP-43^A315T^ mice (N = 8) and Non-Tg littermates (N = 10) with representative images. For statistical analysis, one-way ANOVA with Tukey post-test was performed. **0.001 < *p* < 0.01, ***p < 0.0001. (**C**) Quantification of percentage of GFAP and Iba1 intensity in male symptomatic TDP-43^A315T^ mice (N = 3) and respective Non-Tg controls (N = 7). Comparison between groups independently with unpaired Student’s t test *0.01 < *p* < 0.05, ***p < 0.001. Bar length: 100 μm. (**D**) Quantification of percentage of GFAP and Iba1 signals in female symptomatic TDP-43^A315T^ mice (N = 4) and respective Non-Tg controls (N = 3). Comparison between groups independently with unpaired Student’s t test *0.01 < *p* < 0.05, n.s., *p* > 0.05. Bar length: 100 μm.

Astrogliosis and microglial activation are additional pathological hallmarks of ALS^28,29^. However, neuroinflammation has not been analyzed in spinal cord of TDP-43^A315T^ mice yet. Thus, we performed immunofluorescence staining using anti-GFAP and anti-Iba1 antibodies in the lumbar spinal cord. A significant increase of both GFAP and Iba1 markers was detected in symptomatic TDP-43^A315T^ mice compared to Non-Tg animals (Fig. 4A,B). These differences were observed in both male and female mice (Fig. 4C,D). Importantly, the levels of glial activation observed in mutant TDP-43 mice were comparable to the signal observed in symptomatic SOD1^G93A^ mice. These results indicate that TDP-43^A315T^ transgenic mice develop neurodegeneration and glial activation in the spinal cord, resembling the histopathological changes observed in classical ALS models.

### Aggregation of TDP-43 in the spinal cord and frontal cortex of transgenic mice

**Figure 5:**
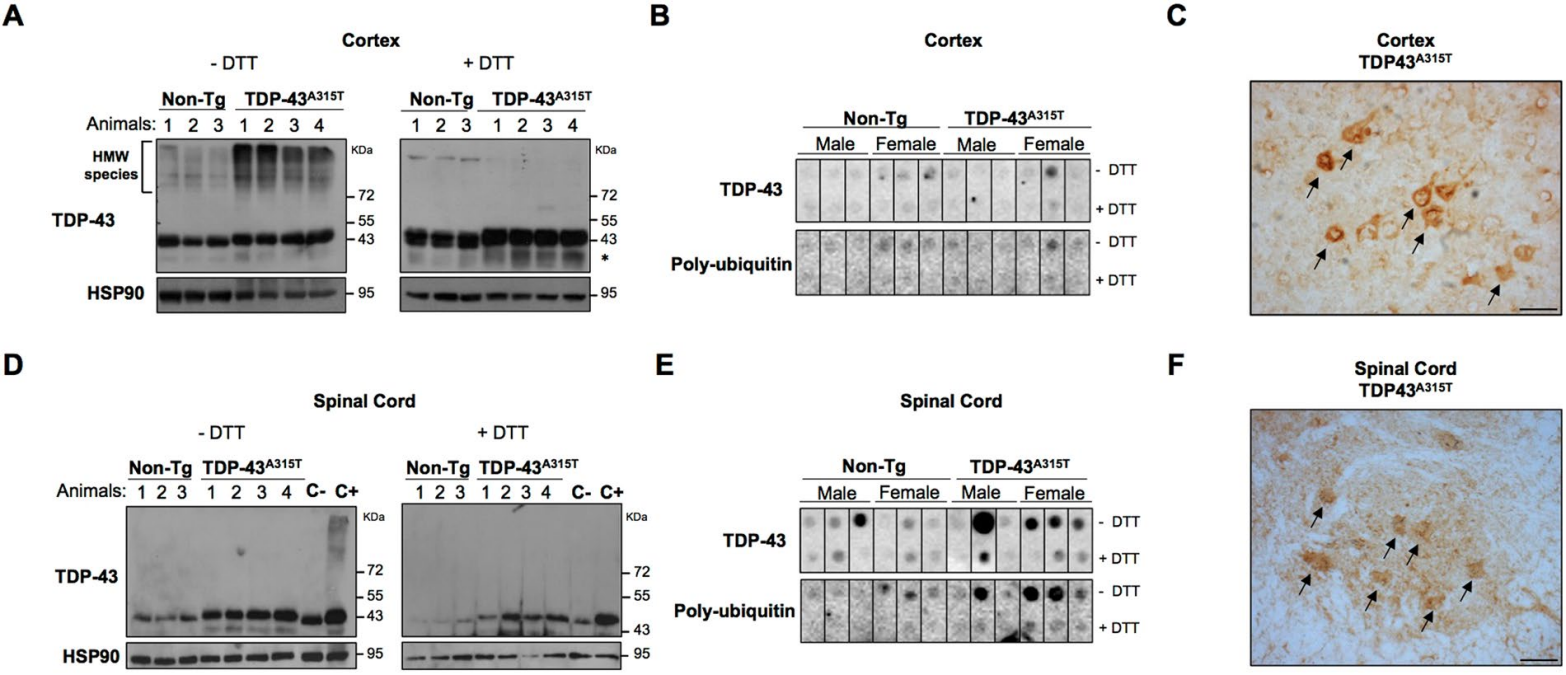
Biochemical analysis of TDP-43 in brain cortex and spinal cord of symptomatic TDP-43^A315T^ mice. (**A**) Detection of TDP-43 protein by Western blot assay. Protein extracts were analyzed in non-reducing conditions (−DTT) and reducing conditions (+DTT, 100 mM) in lysates obtained from cortex of symptomatic TDP-43^A315T^ and Non-Tg mice. *Indicates TDP-43 fragments (**B**) Filter trap assay was applied to assess protein aggregates in brain cortex protein extracts of symptomatic TDP-43^A315T^ and Non-Tg mice. Extracts from male and female mice were analyzed, under non-reducing (−DTT) and reducing conditions (+DTT) for detection of TDP-43 and anti-poly-ubiquitin species. To improve the presentation of this result, cropped images obtained from original membranes are presented. Full-length of all Filter trap membranes are presented in Supplementary Figure 6A. (**C**) Histological analysis of cortical region using an anti-ubiquitin antibody. Positive ubiquitination pattern was detected in pyramidal neurons of the fifth layer of brain cortex of TDP-43^A315T^ mice. Bar length: 40 μm. (**D**) Detection of TDP-43 protein in tissue extracts of spinal cord from symptomatic TDP-43^A315T^ and Non-Tg controls by Western blot. The same experimental approach as (**A**) with lysates in non-reducing conditions (−DTT) or reducing conditions (+DTT). Symptomatic TDP-43^A315T^ brain cortex extracts were loaded as a positive control for aggregation detection (C+) and Non-Tg brain cortex extracts as a negative control (C−). To improve the presentation of this result, a cropped image of the original Western blot is presented. The full-length of this blot is presented in Supplementary Figure 6C. (**E**) Filter trap analysis of TDP-43 and poly-ubiquitinated aggregates in spinal cord extracts from symptomatic male and female TDP-43^A315T^, and Non-Tg littermates. Membranes were blotted under non-reducing (−DTT) and reducing conditions (+DTT). To improve the presentation of this result, crop images obtained from original membranes are presented. Full-length of all membranes are presented in Supplementary Figure 6B. (**F**) Histological analysis of lumbar spinal cord region using anti-ubiquitin antibody. Positive ubiquitination pattern was detected in motoneurons of the ventral horn of TDP-43^A315T^ spinal cord. Bar length: 40 μm. Images of low magnification are presented in Supplementary Figure 7.

A pathological hallmark of ALS and FTD patients is the presence of ubiquitinated TDP-43 protein aggregates in the affected tissues^8^. However, initial characterization of spinal cord and cortical brain tissue failed to detect signs of abnormal protein aggregation in TDP-43^A315T^ mouse^24^. Interestingly, several studies in mutant SOD1 mice have indicated that most protein oligomers and aggregates are formed through disulfide crosslinks^30–32^, which can be detected using standard biochemical methods in the absence of reducing agents. Therefore, we evaluated the presence of oligomers containing TDP-43 in symptomatic TDP-43^A315T^ mice using tissue extracts of frontal cortex and spinal cord by Western blot under in non-reducing conditions. Remarkably, strong aggregation of TDP-43 was observed in cortical tissue of mutant mice (Fig. 5A, left panel). Treatment of protein extracts with the reducing agent DTT fully disassembled TDP-43 aggregates (Fig. 5A, right panel). Surprisingly, these high-molecular-weight species were not observed in spinal cord tissue from the same animals under similar conditions (Fig. 5D). In addition, the presence of TDP-43 fragments were detected on this biochemical analysis. In order to further characterize TDP-43 aggregates, we performed a filter trap assay, which detects large aggregated species over 0.22 μm in size. Surprisingly, we found no retention of TDP-43 protein derived from cortex extracts in the membrane, neither of poly-ubiquitinated proteins (Fig. 5B), suggesting the presence of oligomers rather than high molecular weight aggregates in this brain region. Unexpectedly, we detected large TDP-43 and poly-ubiquitinated aggregates in spinal cord tissue of TDP-43^A315T^ mice using filter trap, which was more consistent in female animals as also observed for motoneuron loss (Fig. 5E). This opposing result of Western blot and filter trap analysis may be due to the fact that large protein aggregates do not enter to the staking gel during the electrophoresis and are lost during Western blot processing. Again, addition of thiol reducing agent to the protein extract (DTT treatment) prevented detection of aggregated species by the filter trap assay. Immunohistochemistry analysis of ubiquitin also confirmed the accumulation of ubiquitinated proteins in both cortex and spinal cord tissue obtained from transgenic animals at symptomatic stage (Fig. 5C and F). These results suggest that different oligomeric and aggregated species of TDP-43 are present in spinal cord and motor cortex of symptomatic TDP-43^A315T^ mice. As previously reported, we did not find the accumulation of large TDP-43 protein inclusions in affected areas of the nervous system as measured by immunofluorescence (Fig. S2). Together, these results demonstrate the presence of abnormal disulfide-crosslinked forms of TDP-43 in affected CNS tissue.

## Discussion

In this study, we have performed global characterization of the transgenic line expressing TDP-43^A315T^ at motor and biochemical levels, and demonstrated that this mouse model recapitulates key disease features. Using different tests we were able to detect the occurrence of progressive motor impairment. Mutant TDP-43 mice also displayed a gender-associated phenotype, consistent with the observation that male ALS patients have a higher risk to develop ALS when compared to women^33^. TDP-43^A315T^ mice develop a complex neurodegenerative phenotype, associated with significant motoneuron loss and axonal degeneration, microglial and astrocyte activation, and abnormal protein aggregation. Importantly, distinct disulfide-crosslinked forms of TDP-43 were found in spinal cord and brain cortex of symptomatic animals.

Overall, our results indicate that the overexpression of human TDP-43^A315T^ causes a progressive motor phenotype. The first description of this mouse model highlighted a “flipper” like motor syndrome^24^ and a following study reported motor impairment^17^. It has been suggested that TDP-43^A315T^ mice die early because of constipation problems^25^, which can be bypassed with fiber-deprived jellified food, expanding the window to observe the degenerative process^34,35^. This intestinal dysfunction is suggested to be a relevant factor contributing to the early death of this transgenic line^34^. However, the fact that we observed significant rates of neuronal and axonal loss, glial activation and motor alterations suggests the occurrence of an active neurodegenerative process. We also found clear differences in the phenotype between genders, where motor alterations and body weight loss occur earlier in male animals. However, both male and female mice, displayed similar duration of the symptomatic phase. Since male and female transgenic mice express similar levels of TDP-43^A315T^, gender-specific factors may explain the differential vulnerability observed at the level of neuronal survival and the progression of disease features. Importantly, we were able to detect robust motoneuron degeneration after performing detailed histological analysis using serial sections, in addition to report evident glial activation to a similar extent as mutant SOD1 mice.

TDP-43 is mislocalized from the nucleus to the cytoplasm in the affected neurons of ALS and FTD patients, observing TDP-43-positive inclusions in a small fraction of neurons. In addition, hyperphosphorylated, ubiquitinated and fragmented forms of TDP-43 are observed in CNS tissue during the disease process^7,8^. Although TDP-43 aggregation is observed in near 97% of ALS cases^36^, the mechanism of action involved in ALS pathogenesis is still not clear. Initial characterization of TDP-43^A315T^ mice failed to detect TDP-43 aggregation, however the presence of large ubiquitin-positive cytoplasmic inclusions was reported in cortical regions^24^. The current study provide evidence indicating that expression of TDP-43^A315T^ under the prion promoter in mice triggers major behavioral and histopathological alterations involved in ALS/FTD, correlating with the accumulation of distinct oligomeric species of TDP-43^A315T^. In addition, the levels of glial activation and the areas of the spinal cord presenting motoneuron loss closely resembled the pathological alterations reported in mutant SOD1 mice, as well as biochemical features including the accumulation of disulfide-crosslinked protein aggregates.

In other neurodegenerative disease, including Alzheimer’s disease, Parkinson’s disease and Huntington’s disease, the accumulation of soluble oligomers has a pivotal role in inducing synapse failure and neuronal dysfunction (review in refs^37,38^). Similarly, studies with ALS-linked SOD1 mutants also suggest that oligomers are highly toxic because they can diffuse easily to reach their molecular targets (see examples in^39–42^). Using different biochemical approaches, we could detect distinct abnormal TDP-43- species in cortex and spinal cord. In particular, we observed TDP-43 oligomers by western blot in brain cortex but not spinal cord extracts, whereas higher-order aggregated species of the protein were detected in spinal cord by filter trap assay. These data suggest that TDP-43 aggregation by disulfide crosslinks is more prominent in spinal cord, possibly contributing to motor problems and motoneurons loss. Although reproducible aggregation of mutant TDP-43 was observed in tissue obtained from end stage mice in our western blot analysis, variability in the levels of high molecular weight TDP-43 aggregates was observed using the filter trap assay. Although we did not explore the biochemical mechanisms explaining these differences it is well-known that both techniques identify distinct oligomeric species and the phenotype associated with larger aggregates may be less penetrant. As described for mutant SOD1-based models, protein aggregates can cause ER stress^43^, protein transport blockage, alter organelle function, inhibition of proteasome machinery, decreased chaperone activity, among others^44^. SOD1-aggregates are formed in part by intermolecular disulfide bonds^45^, which also contributes to the generation of small misfolded oligomers^46^. Since we detected a similar profile of motoneuron loss in TDP-43^A315T^ and mutant SOD1 mice, this may underlie a common mechanism of differential neuronal vulnerability to the disulfide-crosslinked aggregates accumulating of both mouse models. Overall, the data presented here highlights the relevance of the current TDP-43^A315T^ transgenic mice to investigate ALS/FTD pathophysiology, validating this model to test intervention strategies at the preclinical level.

## Materials and Methods

### Colony maintenance and mice monitoring

TDP-43^A315T^ transgenic mice overexpress a mutant form of human TDP-43^24^ mainly in CNS. TDP-43^A315T^ transgenic mice were obtained from the Jackson Laboratory (Strain No. 010700, www.jax.org). For colony amplification and experimental animal generation, breedings were arranged between TDP-43^A315T^ males and Non-Tg (non-transgenic) females on a C57/BL6 pure background. Pups were weaned at age 21 days and, given the aggressive disease phenotype, all mice were monitored at least 3 times a week in order to avoid loss of the colony. The colony was feed with the 5001 LabDiet food, in its pellet form. To monitor disease progression and onset determination, body weight lost was measured and motor performance was evaluated using Hanging and Rota-rod test as reported^47,48^. For TDP-43^A315T^ mice measurements were done until the day of euthanasia. For Non-Tg mice, male and female measurements were made up to 110 days and 160 days of age respectively. Motor test of Rota-rod (Model LE8500, Panlab SL) was performed in TDP-43^A315T^ mice twice a week, starting from the age of 37 days until the day of euthanasia. Accelerated protocol was applied for this motor monitoring, where the rotating wheel increases from 4 to 40 RPM in 2 minutes. Animals were previously trained for three consecutive days and three times per day, first in a constant rate of 4 RPM and then 10 RPM, promoting learning of the task. For Hanging test measurements, mice were placed individually on their front paws on a 39 cm horizontal bar and 35 cm height. Mice were recorded for 30 seconds to follow the behavior and their body position. Each video was analyzed to assign a score and the three measurements per mouse were averaged as described^49^. To assign the score, the following criteria was used: “0”, when the mouse could not hold on the bar more than 10 seconds; “1”, when the mouse just stayed in the bar with the front legs; “2”, when the mouse was able to stay with the front legs and tried to use the rear ones to reach the bar but without success; “3”, when the mouse used the front legs and could use one or two rear legs; “4”, when it used the four paws and the tail; and “5”, where the mouse escaped actively from the horizontal bar and climbed down across the vertical bar in less than 30 seconds. Test was performed once a week, three times each time. The SOD1^G93A^ (Strain No. 010700) was also used, obtained from The Jackson Laboratory.

### Histological analysis of brain tissue

Animals were perfused in a transcardiacal form with 0.9% NaCl and fixed with 4% paraformaldehyde in 0.1 M phosphate buffer (PBS) at pH 7.4. After perfusion, brains were removed and post-fixed on 4% paraformaldehyde overnight at 4 °C. Then brains were dehydrated with 30% sucrose plus 0.02% sodium azide, changing them to the same fresh solution twice every 24 h. Brains were frozen in optimal cutting temperature compound (Tissue Tek) and using a cryostat (Leica) sections of 40 µM thick were prepared. Inmunofluorescence was carried out with standard methods^50^. The brain cuts were incubated with anti-TDP-43 (1:500, ProteinTech Group) on blocking solution overnight at 4 °C. The incubation of slices with secondary antibody was done for 3 h at room temperature (anti-rabbit, 1:1000, Alexa 488) and finally the slices were incubated with the nuclear marker Hoechst (1:5000, Invitrogen) in 1X PBS. Visualization of the slices was done by confocal microscopy using the Olympus IX71 microscope and images were captured with 934 Fast QImaging QICAM. Immunohistochemistry was performed using standards methods, including epitope retrieval incubating slices on 1 M Citrate Buffer for 5 min at 90 °C, washing with 1X TBS and incubating after on 3% H2O2 and 10% Methanol solution for 15 min and wash with 1X TBS. Brain slices were incubated with anti-FLAG (1:250, Sigma) and anti-ubiquitin (1:200, Chemicon) in blocking solution for overnight at 4 °C. After that, slices were incubated with anti-goat-IgG-biotin antibody on blocking solution (1:500, Santa Cruz). Avidin/biotine reaction was performed using a DAB peroxidase Vector Kit (SK-4100). Visualization of the slices was done in a microscope Olympus model IX71 with bright field.

### Histological analysis of spinal cord

Animals were perfused in a transcardiacal form with 0.9% NaCl and fixed with 4% paraformaldehyde in 0.1 M phosphate buffer (PBS) at pH 7.4. For lumbar spinal cord extraction, sciatic nerve was located because it represents the most caudal part of the lumbar section to procedure to make a straight parallel cut. From that reference point another cut was done 5 mm towards rostral direction in order to define our lumbar section. The section was post-fixed on 4% paraformaldehyde overnight at 4 °C. Later, the lumbar spinal cords were incubated on a 7.5% sucrose solution for 1 h, changed after to a 15% sucrose solution for 1 h and then changed again to a 30% sucrose solution overnight at 4 °C. Finally, the sections were embedded on optimal cutting temperature compound (Tissue Tek) and using a cryostat (Leica) slices of 25 µm thick were prepared from caudal to rostral. All the sections were collected individually in 1X PBS 0.1% sodium azide. For stereological analysis, through anti-ChAT immunohistochemistry, 16 slices were selected with a distance of 200 µm each to cover around 3.2 mm of the lumbar section of the spinal cord. Immunohistochemistry was done using standards methods, including epitope retrieval incubating slices on 1 M Citrate Buffer for 5 min at 90 °C, washing with 1X TBS and incubating after on 3% H_2_O_2_ and 10% Methanol solution for 15 min and wash with 1X TBS. Spinal cord slices were incubated with anti-ChAT antibody (1:250, Millipore) in blocking solution for overnight at 4 °C as reported^51^. After that, slices were incubated with anti-goat-IgG-biotin antibody on blocking solution (1:500, Santa Cruz). Avidin/biotine reaction was performed using a DAB peroxidase Vector Kit (SK-4100). Visualization of the slices was done in a microscope Olympus model IX71 with bright field. Inmunofluorescence was carried out with standard methods. The spinal cord cuts were incubated with anti-TDP-43 (1:500, ProteinTech Group), anti-GFAP (1:500, Abcam) or anti-Iba1 (1:500, Wako) on blocking solution, with secondary antibody (anti-rabbit, 1:1000, Alexa 488) and with the nuclear marker Hoechst (1:5000, Invitrogen) in PBS1X. Visualization of the slices was done by confocal microscopy using the Olympus IX71 microscope and images were captured with 1934 Fast QImaging QICAM camera. ImageJ software was used for fluorescent intensity analysis. For cresyl violet staining, 6 slices were selected with 500 µm each to cover around 3.2 mm of the lumbar section of the spinal cord. The slices were stained with 0.5% cresyl violet acetate and visualization of the slices was done in a microscope Olympus model IX71 with bright field.

### Ventral roots quantification

A separate cohort of three transgenic TDP-43 mutant mice and three non- transgenic littermates (controls) at end stage were deeply anesthetized and sacrificed by cardiac transection at end stage. Ventral roots axons were extracted and fixed in 5% buffered glutaraldehyde (pH 7.4) at 4 C for 48 h. Semi-thin cross-sections (1 μm) were stained with toluidine blue, rinsed and coverslipped. Nerve root sections were imaged at 100X magnification as described^52^. Due to low percentage of axonal loss in the transgenic TDP-43 mutant animals (6.13% in TDP-43 mutant animals versus 2.79% in non- transgenic littermates) manual counting was performed of a total of more than 1800 myelinated axons per genotype. No filter was used in the quantification. Axons were defined as conserved or degenerated by the appearance of the myelinated nerve fiber in cross sections. Each category was defined according to the nerve fiber morphologies, as shown in supplementary material (Fig. S5). No evident changes in fiber sizes were found on first inspection. Therefore, the percentage of degenerated axons versus conserved ones was quantified.

### Immunoblotting and filter trap

Cortex and spinal cord extracts were homogenized in 100 µL of 1% PBS Triton X-100 supplemented with a protease inhibitor mix (Roche) and phosphatases inhibitors. For filter-trap and Western blot analysis, brain homogenates were diluted in TEN (10 mM Tris-HCl, 1 mM EDTA, 100 mM NaCl, pH 8.0, supplemented with protease and phosphatase inhibitors mix) buffer containing 0.5% Nonidet P-40 and 50 mM iodoacetamide, sonicated, and quantified. Then, samples were treated or not with the thiol reducing agent dithiothreitol (100 mM DTT) followed by filter-trap and Western Blot analysis as we previously described^53^. PVDF membranes were blotted and probed with either anti-TDP43 (1:3000, ProteinTech Group), anti-polyubiquitin (1:2000, Enzo Life Sciences) and anti-HSP90 (1:3000, Santa Cruz).

### Statistical analysis

Numeric data and quantifications performed for each experimental result were represented on average +/− SEM. Statistics tests used to determine the normality of the data were Kolmogorov-Smirnov, Onmibus D’Agostino & Pearson and Shapiro-Wilk. To determine the differences in data analysis that had a normal distribution, t-Student, one-way ANOVA and two-way ANOVA were used (for multiple comparisons). In the case of data analysis that they did not have a normal distribution, nonparametric Mann-Whitney test was used to detect differences. It was considered with a value of p < 0.05 a difference statistically significant. All these tests were performed using the computational software GraphPad Prism 5.

### Data Availability

All data and associated protocols are available to others without preconditions. The transgenic mice TDP-43^A315T^ that overexpress a mutant form of human TDP-43^24^ can be obtained from the Jackson Laboratory (Strain No. 010700, www.jax.org). The SOD1^G93A^ mice used here correspond to strain No. 010700 and was also obtained from The Jackson Laboratory.

### Compliance with ethical standards

The animal care and all animal experiments were performed according to procedures approved by “Guide for the Care and Use of Laboratory Animals” (Commission on Life Sciences, National Research Council. National Academy Press 1996) and approved by the Bioethical Committee of the Universidad de Chile and from Neurounion Biomedical Foundation (Protocol CBA #05010).

## Electronic supplementary material

Supplementary Information
