# Supplementary material for: Disulfide cross-linked multimers of TDP-43 and spinal motoneuron loss in a TDP-43A315T ALS/FTD mouse model
Source: Sci Rep. 2017 Oct 27;7:14266. doi: 10.1038/s41598-017-14399-5 (PMC5660202; doi:10.1038/s41598-017-14399-5)
Supplement: Supplementary file 1 — Supplementary Information [file 41598_2017_14399_MOESM1_ESM.pdf]

"Disulfide cross-linked multimers of TDP-43 and spinal motor neuron loss in a TDP-43^{A315T} ALS/FTD mouse model"

Leslie Bargsted[2,3,4], Danilo B. Medinas[2,3,4], Francisca Martínez[2,3,4], Pablo Rozas[2,3,4], Natalia Muñoz[1,2,3], Melissa Nassif[5], Carolina Jerez[1,2,3], Alejandra Catenaccio[5], Felipe A. Court[3,5], Claudio Hetz[2,3,4,6,7*] and Soledad Matus[1,2,3,8*].

[1]*Fundacion Ciencia & Vida*, *Santiago,* 7780272, *Chile.*

[2]*Biomedical Neuroscience Institute, Faculty of Medicine, University of Chile, Santiago, Chile.*

[3]*Geroscience Center for Brain Health and Metabolism (GERO), Santiago, Chile.*

[4]*Program of Cellular and Molecular Biology, Institute of Biomedical Sciences, University of Chile, Santiago, Chile.*

[5]*Center for Integrative Biology, Faculty of Sciences, Universidad Mayor, Chile.*

[6]*Buck Institute for Research on Aging, Novato, CA, 94945, USA.*

[7]*Department of Immunology and Infectious diseases, Harvard School of Public Health, Boston MA, USA.*

[8] *Neurounion Biomedical Foundation*, *Santiago, Chile.*

SUPPLEMENTARY MATERIAL

Figure S1. Overexpression of TDP-43 protein in cortex and spinal cord of TDP-43^{A315T} mice. Representative images of FLAG detection of FLAG-tagged TDP-43 by immunohistochemistry in brain cortex and spinal cord tissue of TDP-43^{A315T} mice and Non-Tg littermates in adult stage. For brain cortex, bar length: 270 μm. For spinal cord, bar length: 130 μm.

Figure S2. Overexpression of TDP-43 protein in nervous system of TDP-43^{A315T} mice. The overexpression of TDP-43 using immunofluorescence detection in cortex, hippocampus and spinal cord of TDP-43^{A315T} mice was confirmed using anti-TDP-43 antibody. No TDP-43 inclusions were detected in observed slices. For cortex pictures,

upper bar length: 30 μm, lower bar length: 12 μm. For hippocampus pictures, upper bar length: 150 μm, lower bar length: 25 μm. For spinal cord pictures, upper bar length: 500 μm, lower bar length: 150 μm.

Figure S3. Characterization of the motor phenotype of the TDP43^{A315T} model by the Rota-rod test. The motor test was performed with an accelerated protocol and taking measurements twice a week. Progression curves of each mouse performance were made from the latency time in the rotating cylinder during life. Shown here is an example of how the slope was obtained from progression curves of the TDP43^{A315T} mice (males, left plot; females, right plot). The slope value was calculated from the curve built, taking the measurements from the point of greatest latency time achieved by the mouse and the last measured point prior to its death. For Non-Tg mice, the slope was calculated from the third measurement.

Figure S4. Loss of motoneurons in lumbar spinal cord of symptomatic TDP-43^{A315T} mice. (A) Representative images of ChAT and NeuN immunofluorescence performed in lumbar region of symptomatic TDP-43^{A315T} mice and Non-Tg littermate control for motoneuron quantification. Motoneurons that were positive for ChAT and NeuN were count in 8 slices covering the lumbar region (L3-L5) symptomatic TDP-43^{A315T} mice (N = 4) and respective Non-Tg controls (N = 4). For statistical analysis, two-way ANOVA with Bonferroni post-tests *** $p < 0.001$, * $0.01 < p < 0.05$. Bar length: 100 μm. **(B)** Representative images of macroscopic view of lumbar spinal cord from the ventral horn of TDP-43^{A315T} and control littermates of symptomatic animals. Boxed area is region defined as ventral horn for motor neuron counting. Neurons were count in 6 slices covering the lumbar region (L3-L5) symptomatic TDP-43^{A315T} mice (N = 5) and respective Non-Tg controls (N = 5). Bar length: 300 μm. For statistical analysis, two-way ANOVA with Bonferroni post-tests ** $p < 0.01$, * $0.01 < p < 0.05$.

Figure S5. Motor ventral roots morphology. To determine the content of degenerated axon, a criterion based in nerve fiber morphology was used. Two images of conserved or intact axons (left) are presented. Four representative pictures of the morphologies considered as degenerated axons are shown (right panel). Scale bar: 5 μm. This morphological criterion was applied for quantification of conserved or degenerated axons of ventral roots obtained from L4 and L5 and stained with toluidine blue.

Figure S6. Biochemical analysis of TDP-43 in brain cortex and spinal cord of symptomatic TDP-43^{A315T} mice, full-length membranes (A) Filter trap assay was applied to assess protein aggregates in brain cortex protein extracts of symptomatic TDP-43^{A315T} and Non-Tg mice. Extracts from male and female mice were analyzed, under non-reducing (-DTT) and reducing conditions (+DTT) for detection of TDP-43 and anti-poly-ubiquitin species. Full-length membranes blotted with TDP-43 antibody (left) and poly-ubiquitin antibody (right) (showed in Figure 5B) are shown. **(B)** Filter trap analysis of TDP-43 and poly-ubiquitinated aggregates in spinal cord extracts from symptomatic male and female TDP-43^{A315T}, and Non-Tg littermates. Membranes were blotted under non-reducing (-DTT) and reducing conditions (+DTT). Full-length membranes blotted with TDP-43 antibody (left) and poly-ubiquitin antibody (right) (showed in Figure 5E), are shown. **(C)** Detection of TDP-43 protein by Western blot assay. Protein extracts were analyzed in non-reducing conditions (-DTT) and reducing conditions (+DTT, 100 mM) in lysates obtained from spinal cord of symptomatic TDP-43^{A315T} and Non-Tg mice. Symptomatic TDP-43^{A315T} brain cortex extracts were loaded as a positive control for aggregation detection (C+) and Non-Tg brain cortex extracts as a negative control (C-). An empty lane between spinal cord and cortex samples was left, where molecular weight marker is indicated.

Figure S7. Histological analysis of cortical and lumbar spinal cord region of TDP-43^{A315T} mice using anti-ubiquitin antibody. Histological analysis was performed in both

regions using anti-ubiquitin antibody. Positive ubiquitination pattern was detected in pyramidal neurons of the fifth layer of brain cortex and in motoneurons of the ventral horn of TDP-43^{A315T} spinal cord. Arrows indicate neurons positive stained neurons. For cortex pictures bar length: 40 μm. For spinal cord pictures, bar length: 500 μm.

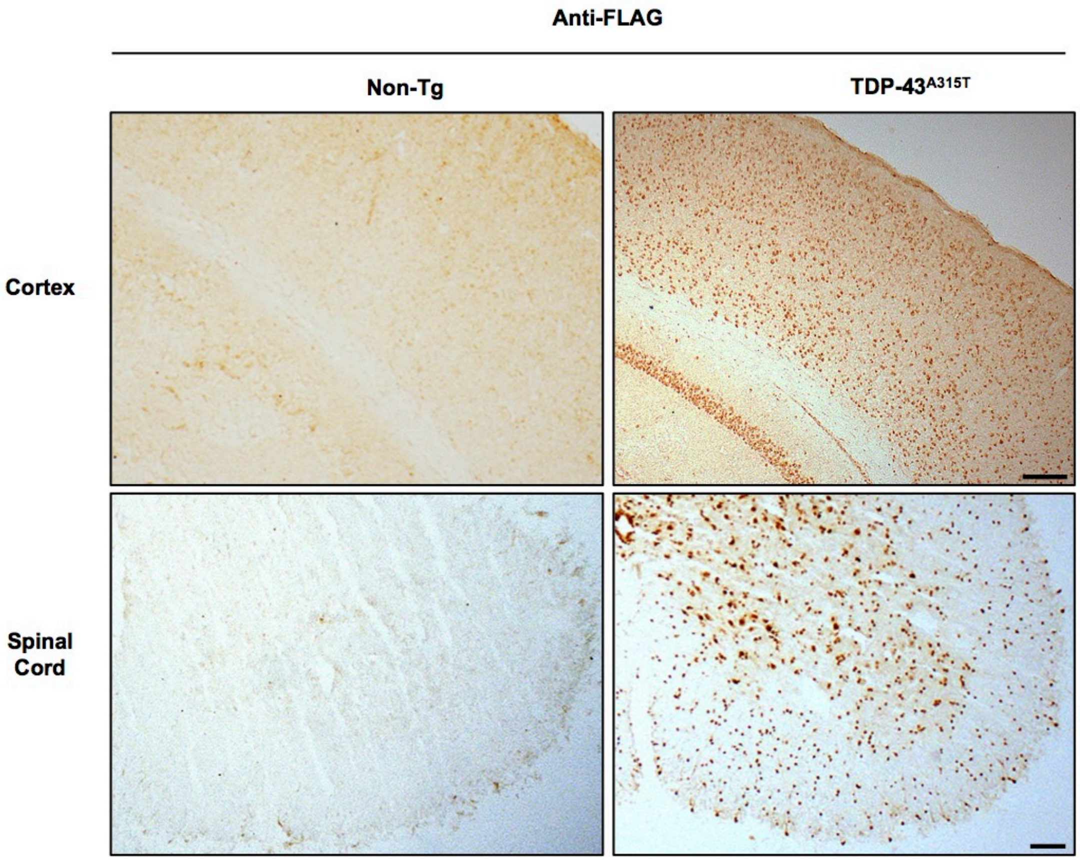

Bargsted et al., Figure-S1

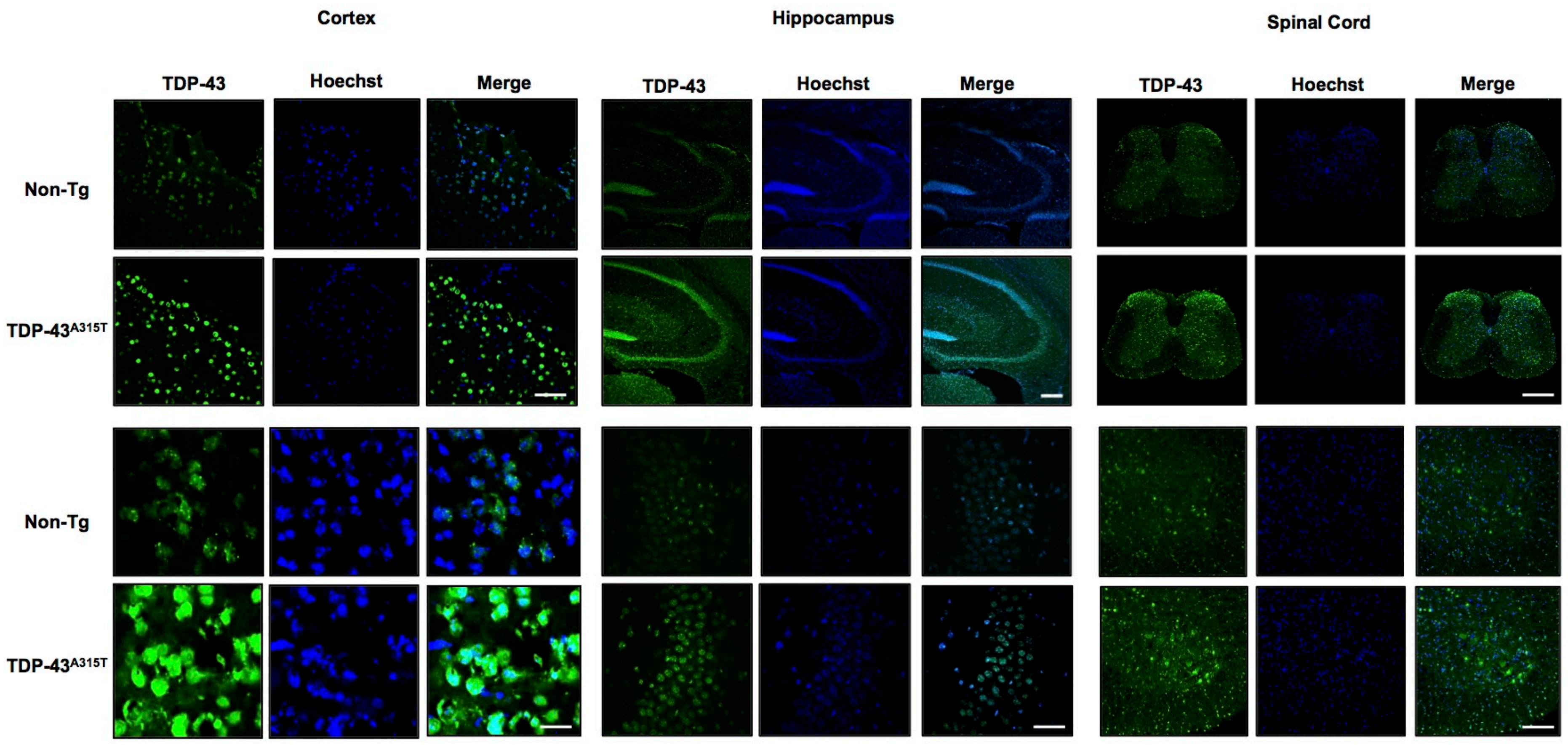

Bargsted et al., Figure-S2

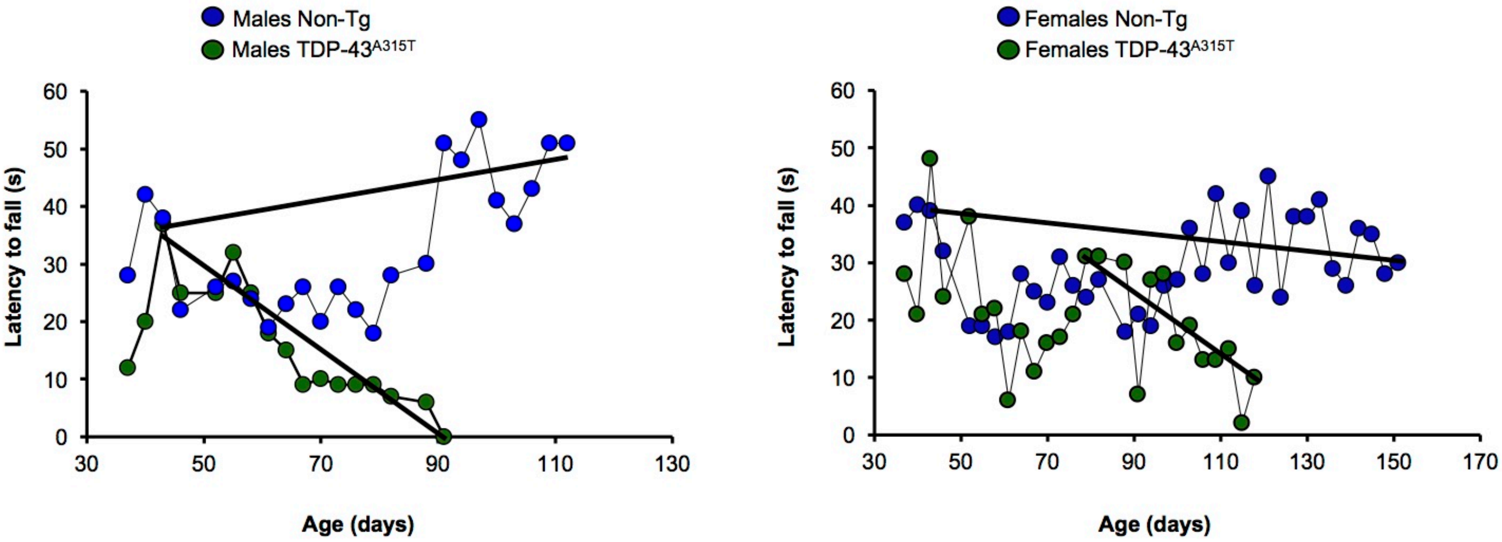

Bargsted et al., Figure-S3

A

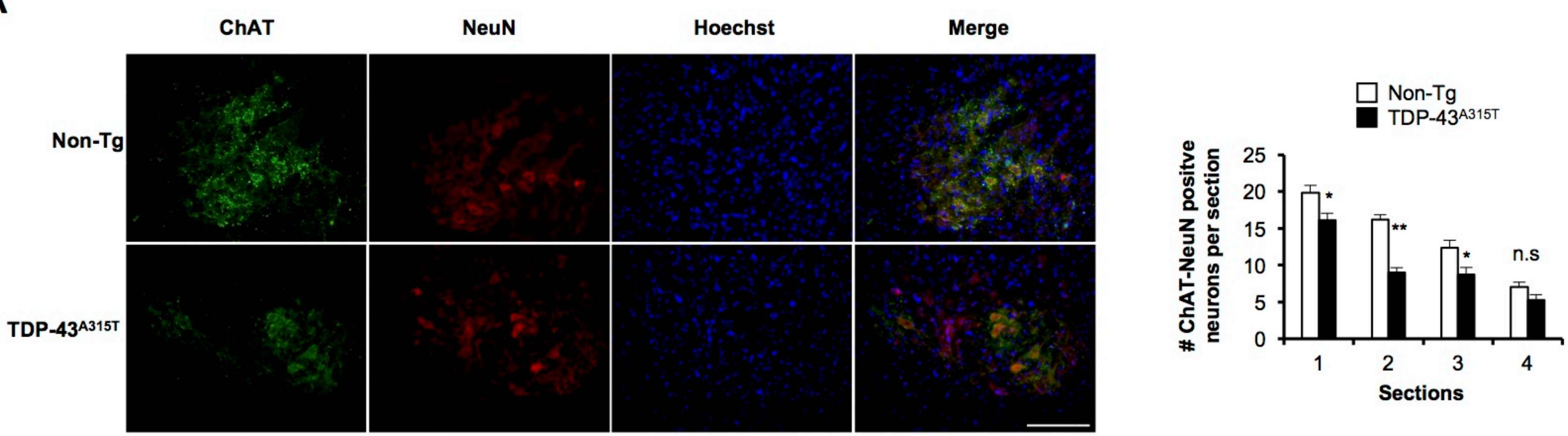

B

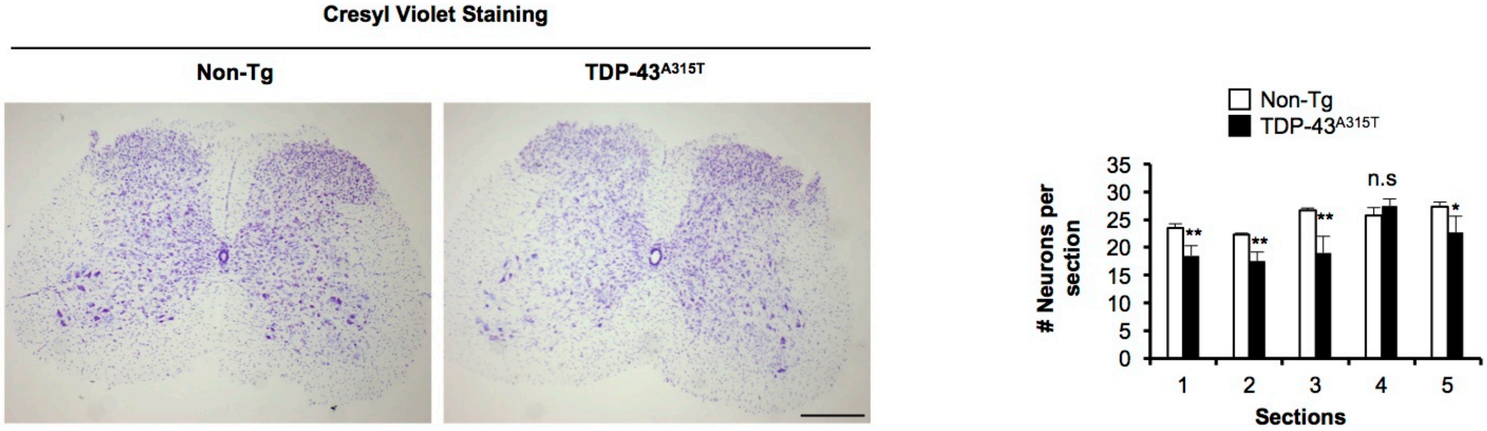

Bargsted et al., Figure-S4

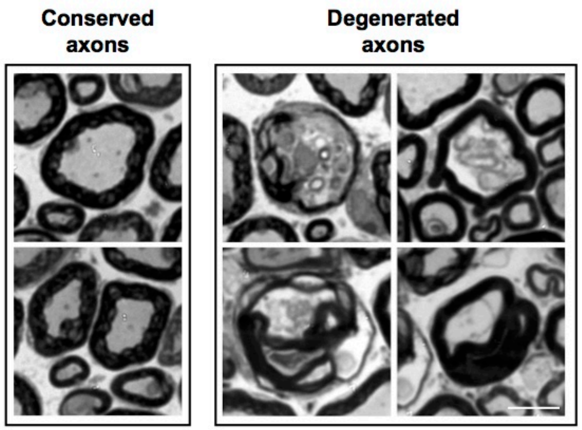

Bargsted et al., Figure-S5

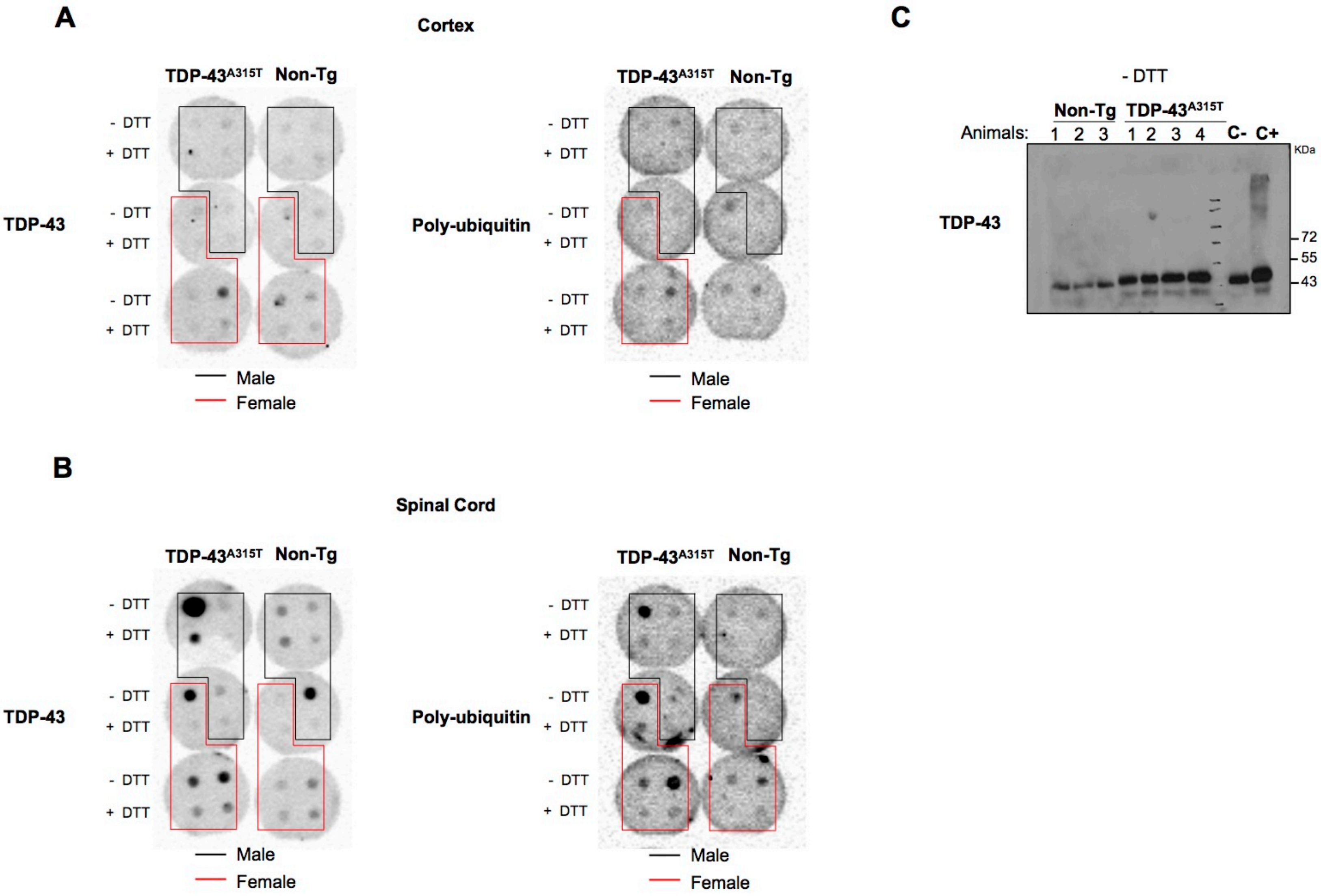

Bargsted et al., Figure-S6

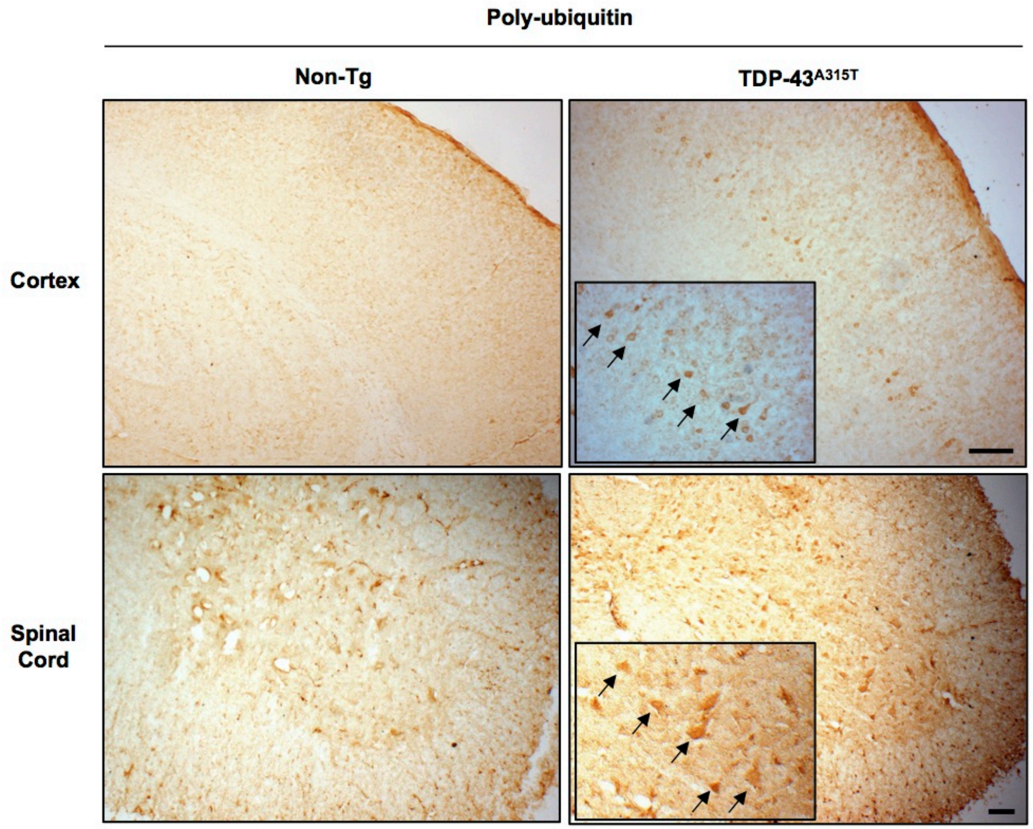

Bargsted et al., Figure-S7
